# Candidemia in Pediatric-Clinic: Frequency of Occurrence, *Candida* Species, Antifungal Susceptibilities, and Effects on Mortality (2020–2024)

**DOI:** 10.3390/diagnostics14202343

**Published:** 2024-10-21

**Authors:** Kamuran Şanlı, Esra Arslantaş, Ayşe Nur Ceylan, Beyza Öncel, Duygu Özkorucu, Ayşe Özkan Karagenç

**Affiliations:** 1 Department of Medical Microbiology, Başakşehir Çam and Sakura City Hospital, University of Health Science, Istanbul 34480, Turkey; aysenurceylan1011@gmail.com (A.N.C.); beyza.asker1@gmail.com (B.Ö.); 2 Department of Pediatric Hematology and Oncology, Başakşehir Çam and Sakura City Hospital, University of Health Science, Istanbul 34480, Turkey; arslesra@hotmail.com (E.A.); duyguozkorucu@gmail.com (D.Ö.); ayseozkankaragenc@gmail.com (A.Ö.K.)

**Keywords:** *Candida*, candidemia, pediatric hematology and oncology, epidemiology, risk factors, *C. parapsilosis* complex, antifungal susceptibility

## Abstract

**Objective:** Invasive candidiasis is defined as an important infection that increases the duration of patients’ hospital stay, costs, mortality and morbidity. In this study, we aimed to investigate the frequency of candidiasis in blood cultures of pediatric hematology patients, *Candida* species, antifungal susceptibilities, and their effects on mortality. **Materials and Methods:** Patients with *Candida* growth in their blood cultures at follow-up in the pediatric hematology clinic of our hospital between 2020 and 2024 were included in the study. Age, gender, primary diseases and risk levels, subtypes and antifungal susceptibilities of *Candida* grown in blood cultures, the presence of neutropenia in patients, the antifungals used for prophylaxis and treatment, the duration of infection, other bacteria grown additionally during the fungal infection period, the local infection source and the patients’ discharge status were obtained from medical records. These constituted the study data. **Results:** Blood cultures were requested for 594 patients from the Pediatric hematology Clinic, and *Candida* was grown in only 37 (6.7%) of them. A total of 43.2% of them were the *Candida parapsilosis* complex, 29.7% were *Candida albicans* and 8.1% were the *Candida haemulonii* complex. Antifungal susceptibilities were over 90% for anidulafungin, micafungin, caspofungin, posaconazole, itraconazole and amphotericin B, followed by 86.7% for fluconazole and 84.4% for voriconazole. The mean age of the patient group was 6.8 years, 50.5% of whom were female and 40.5% of whom were male. The *Candida* infections developed on the 12.1th day of the neutropenia process on average. The mean invasive *Candida* infection period was 7 days. A total of 18.9% had a second bacterial infection and 13.5% had a local infection. A total of 51.4% had a single antifungal, 18.9% had two antifungals and 2.1% had more than two antifungals. A total of 35.1% of the patients with invasive candidiasis died. The primary diagnosis of the disease, Patient risk level, and the female gender were important factors affetting mortality. **Conclusions:** In a pediatric hematology clinic, the non-albicans group in invasive candidiasis infections was notable, with the *C. parapsilosis* complex occurring most frequently. There was still a high sensitivity to echinocandin antifungals and a decreased sensitivity to triazoles. It was found that the factor of the clinical diagnosis, being in the high-risk group and being female had significant effects on the survival rate of patients with candidiasis infections.

## 1. Introduction

The microbiological and clinical features of invasive *Candida* infections vary according to different geographical regions, hospitals, clinics and age [1]. Invasive fungal infections are one of the most important causes of morbidity and mortality in immunocompromised patients. As an opportunistic nosocomial infection agent, *Candida* is increasingly seen in hematological malignant patients due to risk factors such as underlying immunosuppression, intensive antibiotic therapy and steroid use, intravenous catheters, burns, surgical interventions, diabetes mellitus, AIDS and long-term neutropenia due to cytotoxicity [1,2,3,4].

In recent years, the need for antifungal susceptibility tests has increased due to the development of new antifungal drugs with different mechanisms of action that can be used in treatment and increasing antifungal resistance, and antifungal susceptibility tests have started to be used more in clinical laboratories thanks to the developed methods [3].

There are many studies on the frequency and antifungal susceptibilities of *Candida* infections together with different bacteria grown in different body parts in pediatric clinics and pediatric intensive care units in Turkey, along with the risk factors causing *Candida* infections in pediatric hematology-oncology clinics [5,6,7,8]. However, there are very few studies specific to pediatric hematology-oncology clinics on the frequency, antifungal properties and effects on mortality of invasive *Candida* infections grown only in blood cultures. We hope that this study will contribute to the literature regarding Turkish pediatric hematology-oncology.

The aims of our study are to determine the rate of patients with *Candida* growth by examining the four-year blood culture data of patients hospitalized in a pediatric hematology clinic; the frequency of non-albicans *Candida* species by identifying the *Candida species* grown; and the susceptibility profiles of empirically used antifungals by finding the antifungal susceptibilities and MIC value ranges of each *Candida* species.

We also investigated the risk levels of primary diseases, the duration of neutropenia, accompanying local infections and secondary microorganisms, and their effects on mortality in patients diagnosed with invasive *Candida* infections in our hospital’s pediatric hematology clinic.

## 2. Materials and Methods

### 2.1. Study Setting, Data Sources and Ethics

Our hospital (Başakşehir Çam and Sakura City Hospital) is a newly established four-year hospital in Istanbul. During this period (April 2020–April 2024), 594 patients who were hospitalized in the pediatric hematology clinic and whose blood cultures were requested were evaluated, and their files were reviewed using the hospital’s automated system. This study was performed in line with the principles of the Declaration of Helsinki. Approval was granted by the local Başakşehir Çam and Sakura City Hospital ethics committee (28.03.2024 No KAEK/27.03.2024.206). Because of the study’s retrospective nature on deidentified data, informed consent was waived.

### 2.2. Patient Selection

Only patients hospitalized in the Pediatric Hematology Clinic who were diagnosed with invasive *Candida* infections with *Candida* pathogen growth in their blood cultures were included in the study. Growth of the same *Candida* species in blood culture at two different times was considered significant. A total of 37 patients’ data were found to be suitable for evaluation. Patients whose blood cultures did not grow *Candida* were not included in the study. Citizenship numbers were used to ensure uniqueness of patients in the automated system. For each patient with *Candida* growth in their blood culture, the following; were retrospectively examined and recorded separately: age, gender, primary disease of the patient, duration of *Candida* infection, other microorganisms growing in blood culture together with *Candida* infection, presence of local infection, patient mortality and survival status during treatment, antifungal use status and, presence of local infection. If the neutrophil count was below 500 cell/μL, it was considered as neutropenia. If the neutrophil count was above 500 cell/μL, it was not considered as neutropenia. These data were recorded for each patient. In addition, the day on which *Candida* grew in patients with neutropenia was also included in the study. This was ignored for those with a neutrophil count above 500 cell/μL.

Patients who did not have *Candida* in their blood cultures requested by the pediatric hematology-oncology clinic and patients whose results showed repeated *Candida* cultures were not included in the study.

Definition of neutropenia: A decrease below 500 cell/μL is an important risk factor for infection. Therefore, this was determined as the critical value [9].

Infection period: The period of infection was defined as that between the detection of at least one *Candida* species in a patient’ blood culture and the presence of infection and the absence of *Candida* growth in their blood cultures after the treatment process in at least two samples.

High risk/HRG: The treatment protocol of patients presenting to the pediatric hematology-oncology clinic was determined according to the risk categories recommended by the Pediatric Oncology Group (POG) and Childhood Cancer Group (CCG), and the treatment protocols were applied according to this system of categorization. We examined the risk level defined for the primary disease of each patient in the patient files and added these data to our own, dividing the patients into a high-risk group and non-high-risk group.

Invasive Candida infection: Revision and Update of the Consensus Definitions of Invasive Fungal Disease (IFD) from the European Organization for Research and Treatment of Cancer and the Mycoses Study Group Education and Research Consortium was used in neutropenic patients. According to this classification, invasive *Candida* infection is defined as fungus detected by histological analysis or culture of a tissue sample taken from a disease site [10].

### 2.3. Isolation of Clinical Samples

Blood cultures sent for 594 patients were examined in our central laboratory. All patients with *Candida* growth were identified. Only one of the repeated strains isolated from the same patient was included in the study. Blood cultures in BACTEC Plus aerobic medium bottles sent from the pediatric hematology clinic for each patient were incubated in the BACTEC-FX automated blood culture device (Becton Dickinson, Sparks, MD, USA). The media were monitored for five days. During this period, passages were made from the bottles that showed positive growth using 5% sheep blood agar, chocolate agar and eosin-methylene blue agar, and these were incubated at 37 °C for 24–48 h. For the identification of microorganisms grown, at the end of incubation, colony morphology was studied. After identifying *Candida* yeasts by Gram staining, species-level identification was performed using the matrix-mediated laser desorption ionization time-of-flight mass spectrometry method (MALDI-TOF EXS2600 (Zybio, Chongqing, China)

### 2.4. Antifungal Susceptibility Testing

Amphotericin B, caspofungin, fluconazole, flucytosine micafungin, voriconazole, flucytosine and itraconazole were used as antifungals, and their susceptibilities were evaluated using the Colorimetric microdilution method (Thermo Scientific™ Sensititre™ YeastOne™ YO9 AST Plate), (SYO; Thermo Fisher Scientific, Waltham, MA, USA). With this method, the lowest concentration that caused a color change at the end of the 24-h incubation period with 1.5–8 × 10^3^ cfu/mL (0.5 Mc Farland yeast suspension + YeastOne broth (RPMI1640 + 1.5% glucose) inoculation was defined as the MIC value. MIC values were defined as susceptible, dose-dependent susceptible, and resistant according to the recommendations for antifungal agents in the EUCAST guideline (*Candida* and *Cryptococcus* sp. Europen committee on Antimicrobial Susceptibility Testing Breakpoint tables for interpretation of MICs for antifungal agents; version 10.0; valid from 2020 to 2024).

### 2.5. Statistical Method

The data of the study were analyzed using SPSS Version 26.0 statistical package released in 2019; IBM SPSS Statistics for Windows, Version 26.0; Armonk, NY, USA: IBM Corp.). Categorical variables are presented as numbers and percentages, and continuous variables are presented as “mean ± standard deviation” and “median (minimum–maximum)” values. The normality of the continuous data was tested using Shapiro-Wilk test, and since they were not distributed normally, non-parametric tests were used to compare these data between groups. In comparisons between groups, Fisher’s exact chi-square test was used for categorical variables, and Mann-Whitney U test was used for continuous variables. The statistical significance level was accepted as “*p* < 0.05”.

## 3. Results

Blood cultures were requested from the pediatric hematology clinic for 594 patients, and invasive *Candida* infections were grown in only 37 (6.7%) of them. The mean age of the patients was 6.8 ± 4.9 years; 22 (59.5%) were female, and 15 (40.5%) were male. Of these patients, 21.6% had acute lymphoblastic leukemia (ALL), 13.5% had acute myeloid leukemia (AML) and neuroblastoma, 10.8% had B-cell ALL and Ewing sarcoma, 5.4% had rhabdomyosarcoma and osteosarcoma, and 2.7% had recurrent ALL, recurrent AML, T-cell ALL, Wilms’ tumor, and bone marrow failure. The *Candida* infections in the blood cultures developed on the 12.1th day of neutropenia development on average (the earliest was day 0, and the latest was day 75), and the infection duration lasted on average 15.3 (0–42) days.

There were concomitant bacterial infections in seven patients (16.2%); three of these were Gram-negative and three were Gram-positive bacteria. *Staphylococcus haemolyticus*, *Achromobacter xylosoxidans* and, *Pseudomonas aeruginosa* grew simultaneously in the blood cultures of three different patients with complex *Candida parapsilosis* growth, *Staphylococcus epidermidis* was found in the patient with *Kluyveromyces marxianus (Candida kefyr)* growth, *Pseudomonas aeruginosa* was found in the patient with *Candida albicans* growth; and *Enterococcus faecalis* was found in the patient with *Nakaseomyces glabrata (Candida glabrata)* growth. Five patients had local infections (13.5%). While seven patients (18.9%) did not use antifungals, 51.4% used one antifungals, and 8.1% used more than two antifungals. The most commonly used antifungal treatment was single caspofungin (28.0%), followed by fluconazole (18.9%). Fluconazole and caspofungin were used as a dual therapy in three cases; fluconazole and amphotericin B were used in two cases, and triple antifungals were used in three patients (Table 1).

The distribution of *Candida* species grown in the blood cultures in the microbiology laboratory was as follows: 16 (43.2%) out of 37 patients had a *Candida parapsilosis* complex, 11 (19.1%) had *Candida albicans*, three (8.1%) had a *Candida haemulonii* complex and, two (5.4%) had *Candida tropicalis*. *Nakaseomyces glabrata*, *Kluyveromyces marxianus*, *Pichia kudriavzevii* (*Candida krusei*), *Clavispora lusitaniae* (*Candida lusitaniae*), and *Candida* spp. (2.7%) were detected in one patient each (Table 1).

When the mortality rates of patients with *Candida* infections were examined, it was found that 55% of the female patients, 15.4% of the male patients, 78.4% of the patient group under 10 years of age and, 21.6% of the patient group over 10 years of age died. A total of 58.8% of those whose primary disease was HRG, 18.8% of those without HRG, and 44.8% of the patient group with neutropenia died. All of the non-neutropenia group survived. The mortality rates of those with the simultaneous growth of other microorganisms and *Candida* infections and those with local infection were 50% and 40%, respectively (Table 1).

When the antifungal susceptibilities of all *Candida* species were evaluated, all *Candida* isolates were found to be susceptible: 100% were susceptible to micafungin and caspofungin, 94.3% were susceptible to anidulafungin, 93.3% were susceptible to posaconazole, 86.7% were susceptible to voriconazole, 96.4% were susceptible to itraconazole, 84.4% were susceptible to fluconazole, and 97.1% were susceptible to amphotericin B. The MIC_50_ and MIC_90_ values of all antifungals were 0.15 µg/mL and 2 µg/mL, respectively. Those of each individual antifungal were as follows: anidulafungin; micafungin: 1 µg/mL and 2 µg/mL; caspofungin: 0.25 µg/mL and 0.5 µg/mL; posaconazole: 0.015 µg/mL and 0.06 µg/mL; voriconazole: 0.015 µg/mL and 0.5 µg/mL; itraconazole: 0.06 µg/mL and 0.12 µg/mL, fluconazole: 0.5 µg/mL and 128 µg/mL; and; amphotericin B: 0.5 µg/mL and 1 µg/mL (Table 2).

Since EUCAST has not defined a susceptible resistant threshold for *Candida* for flucytosine, only MIC_50_ and MIC_90_ values were determined. The border range was ≤0.6 µg/mL–≥0.12 µg/mL and <0.06 µg/mL–1 µg/mL, respectively.

When the antifungal susceptibilities of the most common *Candida* species were examined, all *Candida parapsilosis* complex (16 patients) isolates were susceptible (100%) to anidulafungin, micafungin, caspofungin and itraconazole. Susceptibility rates to posaconazole and amphotericin B were 93.8%, and 81.3% of the isolates were susceptible to fluconazole.

*Candida albicans* was isolated from 11 patients. Of these isolates, 90.9% were susceptible to anidulafungin and all (100%) were susceptible to other antifungal drugs.

All *Candida haemulonii* complex strains (three patients) were susceptible to anidulafungin and amphotericin B, and since no breakpoints were defined for other antifungal agents according to EUCAST, MIC_50_ and MIC_90_ values were defined. The values are shown in Table 2.

The two *Candida tropicalis* isolates were both susceptible to fluconazole and amphotericin B. The susceptibility rate for these two isolates to each of the other antifungal agents (except flucytosine) was 50%.

*Kluyveromyces marxianus*, *Nakaseomyces glabrata*, *Pichia kudriavzevii* and *Candida* spp. grew in only one patient each. In the EUCAST guidelines for evaluating antifungals, the following did not have defined suspectibility and resistance breakpoints: all antifungal drugs for *Kluyveromyces marxianus*; flucytosine, posaconazole, voriconazole and, itraconazole for *Nakaseomyces glabrata*; and micafungin, flucytosine, posaconazole, voriconazole and, itraconazole for *Pichia kudriavzevii*. Therefore, they could not be evaluated. Other defined antifungal drugs were susceptible to these *Candida* species. Only *Nakaseomyces glabrata* and *Pichia kudriavzevii* were found to be resistant to fluconazole.

Table 2 shows the MIC_50_ and MIC_90_ values of the antifungal drugs used against the most common *Candida* species.

## 4. Discussions

In this study, *Candida* species were grown in the blood cultures of 37 patients, of whom 22 (59.5%) were female and 15 (40.5%) were male, with a mean age of 6.8 ± 4.9 years, who were hospitalized in the pediatrichematology-oncology clinic. Their antifungal susceptibilities and the effects of these factors on the death of the patients were investigated.

Multicenter prospective studies of pediatric healthcare-associated bloodstream infection in the United States and Europe identify pediatric and neonatal invasive *Candida* infections as the third most common cause [11,12]. The duration of neutropenia and absolute neutrophil count are important factors affecting the risk of infection in neutropenic patients [13,14]. A multicenter retrospective study in children reported that an absolute neutrophil count (ANC) of ≤500 cells/μL at the beginning of a chemotherapy cycle increased the probability of IFD by three-fold. In these patients, the infection progresses with vague symptoms because of impaired host defenses [13,14]. This makes it difficult to identify the infection. Fewer studies have been conducted on neutropenic children compared to adults. This rate varies between 40% and 60% in different studies [5,13]. In our study, neutropenia (ANC ≤ 500 cell/μL) was seen in 88.6% of the patients who developed *Candida* infection, compared with 16.2% in those without neutropenia (ANC ≥ 500 cell/μL). *Candida* grew for an average duration of 12.1 days in patients with neutropenia. The rate of candidemia in those with neutropenia is higher than the rate defined in the literature. Sezgin Evim et al. [6] defined this period as 16 days in their study. In our study, 44.8% of patients with neutropenia recovered and were discharged, while 55.2% died. In patients who did not develop neutropenia (16.2%), no one died, and 100% recovered and were discharged. No significant result was found in the evaluation of the relationship between neutropenia and death (*p* > 0.05). In our country, in their study titled “Mortality Risk Factors for Invasive Candidiasis in the Pediatric Intensive Care Unit”, Korulmaz et al. [7] found a relationship between leukopenia, thrombocytopenia and mortality (*p* < 0.05), but although they investigated neutropenia among the risk factors in their table, they did not define it among the factors affecting mortality. However, neutropenia, a serious side effect of chemotherapy, has been identified in studies as an important factor that increases mortality [13,14,15].

In our study, of the patients who were hospitalized in the pediatric hematology clinic who had developed candidemia infections, 21.6% had ALL, 13.5% had AML and neuroblastoma, 10.8% had B-cell ALL and Ewing sarcoma, 5.4% had rhabdomyosarcoma and osteosarcoma; and 2.7% had relapsed ALL/AML. Among all the patients who developed candidemia infections, the group diagnosed with ALL was the largest (21.6%). In the analysis performed by correlating the effect of the primary disease diagnoses of the patients and the association of candidemia infection on survival, a statistically significant difference was found between the groups; the mortality rates of patients diagnosed with AML and Ewing sarcoma were found to be lower (*p* < 0.05). In their study comparing children with AML and ALL leukemia, Sezgin Evim et al. [6] reported that invasive fungal infection had a higher mortality rate in children with ALL and defined the mortality rate of invasive *Candida* infection as 24%. Many studies report that the prevalence of invasive *Candida* infection in patients with hematological malignancies ranges from 4% to 35%, depending on the chemotherapy protocol used, existing risk categories, and the antifungal prophylaxis used [6,7,8,9,10,11,12,13,14,15,16]. In our study, it was most frequently seen in patients with ALL, and the total mortality rate was 35.1%. When the patients were evaluated according to their clinical stages, 48.6% of our patient groups were in the high-risk group (HRG), and 51.4% were not in the high-risk group (NonHRG). A total of 58.8% of the HRG group died and 41.2% survived. The mortality rate in the NonHRG group was found to be 18.8%. In the analysis of survival among patients with candidemia of the disease stage, a statistically significant difference was found (*p* < 0.05). This suggests that the stage of the primary disease is an important factor in deaths from *Candida* infection, and *Candida* infection is more fatal in the high-risk group due to more aggressive treatment.

In our study, six of the patients had concomitant bacterial infections; three of these were infections with Gram-negative bacteria and, three with Gram-positive bacteria. Candidemia attacks caused by more than one microorganism (bacterial-fungal) are important because they are associated with a-higher mortality rate. According to information in the literature, the frequency of bacterial-fungal co-growth is between 2.8% and 8.0% [17]. In a study conducted in our country, this rate was reported as 8.7% [8]. In our study, this rate was found to be higher at 16.2%. When the effects of *Candida* and secondary microorganisms on patient mortality were evaluated, no significant results were obtained.

According to information in the literature, invasive candidiasis can spread to many organs. In immunocompromised patients, it is recommended to evaluate for metastatic foci in the brain, eye, lung, liver, spleen, kidney and central nervous system [18]. In our study, among the patients with candidemia in their blood culture, only five patients (13.5%) had a local infection. Two patients had a lung infection. *Candida* does not infect the respiratory tract after inhalation as does mold fungi. *Candida* spp. colonize the upper respiratory tract and are usually identified in sputum or deep lung cultures, suggesting colonization rather than invasive infection. Hematogenous disseminated pulmonary candidiasis can be identified by chest computed tomography, presenting as pulmonary nodules [18]. The others were hepatosplenic candidemia, scrotal lesions and skin infections on the back. Hepatosplenic candidiasis (HSC) has been defined as a rare infection that occurs in hematological malignant patients with prolonged neutropenia and has been reported in patients with AML at rates ranging from 3% to 29%. It was reported that *C. albicans* was the most common causative agent [19]. In our study, similar to the literature, the primary diagnosis of the patients was AML, and *C. albicans* was grown in their blood cultures.

When the effects of *Candida* infection and concurrent local infection on patient mortality were examined, no significant results were obtained. (*p* > 0.05).

The majority of patients receiving inpatient treatment in the clinic received single antifungal therapy (51.4%), 18.9% received dual-antifungal therapy, and 8.1% received multiple antifungals. IDSA guidelines for candidemia recommend initial treatment with echinocandin [20]. In our study, the most commonly used agent was echinocandin. Of the echinocandins selected prophylactically, 10 were caspafungin and two were voriconazole. Fluconazole was preferred in only five patients. Lysosomal amphotericin B was used in one patient with a neuroblastoma diagnosis. Second and third antifungals were added according to the patient’s infection parameters and additional pathologies. Antifungals were not used in seven of them. Three of the patients who did not use antifungals were in the high-risk group and had growth at day 0 of neutropenia. All three died due to metastatic progressive primary disease. One had growth in his blood culture after being discharged at his own request, and there was no follow-up. In the other three patients, prophylaxis was not started because the neutrophil count did not fall below 500 cells/μL. Since the cause was thought to be catheter-related, the culture after catheter removal was negative. The period of the candidiasis was an average of 7 days in the discharged patients and 9 days in the group of patients who died. When the relationship between the antifungal used in treatment and mortality and the relationship between the duration of infection and mortality were examined, it was observed that their effects on death were not significant (*p* > 0.05).

When patients with *Candida* growth in blood cultures were evaluated by age group, the mortality rate was found to be 34.6% in the 0–10 age group and 57.1% in the over-10 age group. No statistically significant difference was found between the groups in terms of survival status according to age group (*p* > 0.05). Some studies suggest that ages over 9–10 years are associated with higher rates of invasive fungal infections [6,16,21]. There are studies reporting that more aggressive treatments are applied in this patient group, that invasive fungal infections may be seen more frequently, and that age alone cannot be a parameter [16]. In our study, 14 of 30 patients in the 1–10 age group had primary diseases in the high-risk group, and seven of them died. The number of patients over 10 years of age was seven, five of whom were in the high-risk group and three of whom died. In our study, *Candida* infection was more common in the group younger than 10 years of age and more fatal in the group over 10 years of age. The fact that it was more lethal in those over 10 years of age suggested that it might be related to the application of a more aggressive treatment protocol in this age group.

Blood cultures requested from 594 patients were included in the study. *Candida* growth occurred in 6.7% of the blood cultures. Of these *Candidas*, 43.2% were a *C. parapsilosis* complex, 19.1% were *C. albicans*, 8.1% were a *C. haemulonii* complex, 5.4% were *C. tropicalis* and 2.7% were *N. glabrata*, *K. marxianus*, *P. kudriavzevii*, *C. lusitaniae* or *C.* spp. (each of which infected one patie). In comparison to studies conducted in our country; in a study examining the frequency of *Candida* in the blood culture of 75 febrile neutropenic patients, the frequency of fungal infection in blood cultures was 8.8%. In another center where 78 patients were evaluated, the frequency of fungal infection in blood cultures was 9% in pediatric hematology-oncology patients. And in another study conducted on 342 patients hospitalized in the pediatric oncology ward, the frequency of fungal infection was 11% [22,23,24]. In a study conducted by Bektaş et al. [25] on 63 patients in pediatric hematology-oncology clinics, 55.75% had *C. albicans*, 13.27% had a *N. glabrata* and *C. parapsilosis* complex, 12.39% had *C. tropicalis,* 6.19% had *K. marxianus,* 2.65% had *P. kudriavzevii* and 0.88% had *C. guilliermondii*, *C. lipolytica* or C. lusitaniae. In the study by Çağan et al. [26], the cause of invasive *Candida* infections in blood cultures was identified as *Candida albicans* in 55.1% of cases, a *C. parapsilosis* complex in 27.5% of cases, and *C.guillermondii*, *N. glabrata*, *C. tropicalis* and *P. kudriavzevii* in 6.8% of cases.

In studies conducted abroad, in India, where 2198 blood cultures were examined, 4.7% of pediatric patients in a tertiary cancer center were diagnosed with *Candida* [27]. In a study conducted by Müllen et al. [28] on 38 pediatric cancer patients, non-albicans *Candida* fungemia was more common and was identified as *C. albicans* in 29% of cases, *C. tropicalis* in 26% of cases, *C. parapsilosis comple* in 24% of cases, *P. kudriavzevii* in 8% of cases, *N. glabrata* in 8% of cases and *C. lusitaniae* in 5% of cases. Abdel-Hamid et al. [29] also showed that non-albicans *Candida* infections were found at a high rate in cancer patients and that infections due to *C. tropicalis* species were significantly associated with hematological malignancies. In addition, Fisher et al. [12] reported in a study that the frequency of non-albicans species increased after 1990 and that all *Candida* species caused 35–65% of candidemia in the general population. They reported that they were more frequently seen in cancer patients, especially in patients with hematological malignancies and bone marrow transplant (BMT) recipients (40–70%), and defined the frequencies as follows: the *C. parapsilosis* complex (20–40% of all *Candida* species), *C. tropicalis*: 10–30%, *P. kudriavzevii:* 10–35%, *N. glabrata:* 5–40%, *C. lusitaniae:* 2–8% and *C. guilliermondii:* 1–5%, respectively. In addition, Vasileiou et al. [30] reported that in their study examining 4-year patient data from a pediatric hematology-oncology clinic in Greece, candidemia was detected in 19 patients, with the *C. parapsilosis* complex being the most common and *C. albicans* being the second most common. Our study, unlike other centers in our country, showed that invasive *candida* infection was less common in pediatric hematology-oncology patients. Also, non-albicans *Candida* species were isolated more frequently from blood cultures. Our study is similar to other countries in the world in terms of the frequency of non-albicans candidemia. *C. tropicalis* is described in some sources as being more common in patients with hematological malignancies [9]. In our study, it was observed to be the third most common non-albicans *Candida* infection. *C. parapsilosis* complexes were the most common causative agent, as reported by Vasileiou et al. [30]. Many sources describe that infections caused by *C. parapsilosis* complexes in hospital environments, can be transmitted through the hands of healthcare workers and contaminated medical devices, leading to hospital outbreaks [31,32]. This difference observed between species in our study may vary according to geographical regions and the source of the infection, and suggests that infection control efforts should be more thorough. 

Studies have shown that non-albicans invasive *Candida* infections are increasing, and the identification of increasingly resistant *Candida* strains causes concerns about treatment success. Therefore, it is recommended that each center conduct studies that will facilitate the initiation of effective empirical treatments with prophylactics [5,6,28,29]. Azoles, echinocandins and amphotericin B are frequently used in *Candida* infections. Triazoles are the most commonly used antifungal agents due to their high oral bioavailability and general safety. Their frequent use in both preventive and experimental treatment has also led to the problem of an increase in azoles-resistant *Candida* species [33,34].

In a study investigating the resistance mechanisms of invasive *Candida* agents with the participation of 60 countries worldwide, Castanheira et al. [34] defined more than 90% of *C. albicans* strains as susceptible to azoles; 89.1–91.6% of *C. parapsilosis* complex isolates were found to be susceptible (the most resistant isolates were found in Italy in Europe (15.1%); resistance to fluconazole and voriconazole was detected in 0.4% and 0.1% of *C. albicans* isolates, respectively; fluconazole resistance was detected in 6.5% of *N. glabrata* (the highest resistance was in the USA, 13%); and resistance to voriconazole in *Pichia kudriavzevii* was reported only in the USA (5.0%).

In a study evaluating the clinical samples of *Candida* grown between 1997 and 2017, in which 12 third-level hospitals participated in our country, it was reported that no fluconazole resistance was detected in *C. albicans* isolates, fluconazole resistance was detected in *C. parapsilosis* complexes (7.7%), and fluconazole resistance was detected in *N. glabrata* (0.9%) [35]. Another study, drawing attention to itraconazole resistance (41.7%) among *Candida* species, reported resistance to fluconazole (7.4%) and resistance to voriconazole (9.1%) in samples in which *C. albicans* was detected [36].

In our study, it was observed that azole antifungals had reduced levels of susceptibility compared to other antifungals in all *Candida* species. Fluconazole, voriconazole, posiconazole and itraconazole were detected to have sensitivities of 84.4%, 86.7%, 93.3% and, 96.4%, respectively, and the most reduced susceptibility was found for fluconazole and vorconazole. Their MIC_50_ and MIC_90_ values were remarkable: 0.50 µg/mL–128 µg/mL and 0.015 µg/mL–0.50 µg/mL, respectively. At the species level, the least susceptible group to azoles was *C. parapsilosis* complex strains, showing susceptibilities of 81.4%, 86.7%, 93.3% and 100%, respectively, and decreased levels of susceptibility to fluconazole and voriconazole, with MIC_50_ and MIC_90_ values of 0.5 µg/mL–32 µg/mL and 0.015 µg/mL–0.5 µg/mL, respectively. *C. albicans* strains were found to be susceptible to all azole groups. There were only two *C. tropicalis* strains and one strain was susceptible. *C. haemulonii* complexes grew three strains and *N. glabrata* was susceptible to all azoles except fluconazole. *P. kudriavzevii* was found to be susceptible. Other studies were found to be similar to our study, with *C. albicans* being 100% susceptible and non-albicans *candida* having reduced levels of azole susceptibility.

Amphotericin B is a broad-spectrum antifungal. It increases its fungicidal effect according to the blood concentration level. Therefore, it is an agent with s strong post-antifungal effect. Its mechanism of action is by binding to ergosterol in the fungal cell membrane and increasing cell permeability [33].

There are many publications in the world and in our country reporting the high level of sensitivity of *Candida* species to amphotericin B [33,34]. In our study, a 97.1% sensitivity to amphotericin B was found. Only one isolate of the *C. parapsilosis* complex was found to be resistant (6.2%). This shows that is still important in the treatment of invasive *Candida*. The high sensitivity to amphotericin B in our clinic may be due to the fact that it is not the first-choice antifungal agent for treatment or as a prophylaxis.

Since the echinocandin group of drugs cannot enter the fungal cell, they are not affected by resistance mechanisms developed against azoles, such as changing the sterol structure or creating an exclusion pump [33]. Castanheira et al. [34] reported low resistance rates to echinocandin antifungal drugs and reported that *Candida albicans* isolates were resistant to andilofungin, caspafungin and, micafungin (0.1%, 0.2% and, 0.2%) (most commonly found in Europe), the *C. parapsilosis* complex and *P. kudriavzevii* were not resistant, and *N. glabrata* was resistant (2.3%, 1.7% and, 1.7%). In a study conducted by Arikan-Akdagli et al. [35] in 12 centers, they reported that there was no echinocandin-resistant *Candida*. In our study, only two of all of the *Candida* isolates were found to be resistant to anidulafungin, one of which belonged to *C. albicans* and the other to *C. tropicalis*. The low rate of echinocandin resistance suggests that invasive *Candida* can be used as an alternative antifungal agent in cases of azole resistance. Therefore, monitoring and reporting of antifungal susceptibility data are extremely important [5,15,16,22,30,33]. The susceptibility profiles of non-albicans *Candida* species and other less common yeasts may help clinicians make better decisions regarding patient treatment. We believe that this study contains important data for our hospital’s pediatric hematology-oncology clinic.

In our result, candidemia was seen at a high rate in neutropenic hematology patients and its relationship with mortality was not found to be statistically significant. It was observed that the primary disease of the hematological malignant patient group and the disease being in the HRG group had a statistically significant effect on the survival rate of patients with candidemia infections. Although there was no statistically significant difference in survival between age groups, the mortality rate was higher in pediatric patients over 10 years of age. This situation suggested that the effect of candidemia infection on mortality is related to the risk level of the patient’s primary disease and the aggressive chemotherapy used. The results obtained with 37 patients in our clinic cannot be generalized, and more multicenter studies are required on this subject.

*Candida* was grown in 6.7% of all pediatric hematology oncology clinic patients whose blood cultures were requested, and although this rate was lower than that in studies in our country, non-albicans *Candida* species were more common. Among non-albicans *Candida*, the most common hospital-acquired opportunistic pathogen known as the *C. parapsilosis* complex was the most common pathogen in our study. This drew attention to the importance of complying with hospital control recommendations.

In addition, it was observed that sensitivity to azole antifungal drugs decreased in the groups, and echinocandins are an important alternative for patient prophylaxis. In our clinic, echinocandin antifungals are the first drug group selected. It is recommended that each center defines its own culture and antifungal susceptibilities and that treatment regimens should be arranged accordingly.

## 5. Limitations

Our study has several limitations. It is a retrospective study. It is limited to a single center. A total of 37 *Candida* species were grown in the blood cultures of 594 different patients in 4 years. The sample size is limited. No comparison was made between patients with and without candidemia. Classic risk factors evaluated for candidemia were not investigated.

## 6. Conclusions

It is recommended that the relationship between the risk levels of primary diseases and candidemia in pediatric hematology-oncology patients be reviewed in multicenter studies. Each center should determine the most common candida agent in its clinic and design an antifungal treatment protocol.

## Figures and Tables

**Table 1:** Basic descriptive characteristics and survival status of patients with *Candida*.

	All Patiens (*n* = 37)	Survival Status		*p*
		Died (*n* = 13)	Discharged (*n* = 20)	
**Age** mean ± SD; median (minimum–maximum)	6.8 ± 4.9 5.0 (1–19)	7.7 ± 6.3 5 (1–19)	6.6 ± 4.1 5 (2–18)	0.985 *
**<10 Age**	29 (78.4)	9 (34.6)	17 (65.4)	0.393 **
**≥10 Age**	8 (21.6)	4 (57.1)	3 (42.9)	
**Gender *n* (%)**				
Female	22 (59.5)	11 (55.0)	9 (45.0)	0.032 **
Male	15 (40.5)	2 (15.4)	11 (84.6)	
**Diagnostic data *n* (%)**				
ALL	8 (21.6)	-	7 (100.0)	0.041 **
ALL/relapse	1 (2.7)	1 (100.0)	-	
AML	5 (13.5)	1 (20.0)	4 (80.0)	
AML relapse	1 (2.7)	1 (100.0)	-	
AML-down	1 (2.7)	-	1 (100.0)	
B cell ALL	4 (10.8)	-	3 (100.0)	
Evans/hemolitic anemia	1 (2.7)	1 (100.0)	-	
Ewing sarcoma	4 (10.8)	1 (25.0)	3 (75.0)	
Bone marrow failure	1 (2.7)	1 (100.0)	-	
Neuroblastoma	5 (13.5)	3 (60.0)	2 (40.0)	
Osteosarcoma	2 (5.4)	1 (100.0)	-	
Rhabdomyosarcoma	2 (5.4)	2 (100.0)	-	
T cell ALL	1 (2.7)			
Wilms’ tumor	1 (2.7)	1 (100.0)	-	
**Risk group *n* (%)**				
HRG ***	18 (48.6)	10 (58.8)	7 (41.2)	0.032 **
NonHRG ****	19 (51.4)	3 (18.8)	13 (81.2)	
***Candida* Species *n* (%)**				0.239
*Candida parapsilosis*	16 (43.2)	3 (21.4)	11 (78.6)	
*Candida albicans*	11 (29.7)	5 (50.0)	5 (50.0)	
*Candida haemulonii*	3 (8.1)	1 (33.1)	2 (66.7)	
*Candida tropicalis*	2 (5.4)	1 (100.0)	-	
*Candida glabrata*	1 (2.7)	1 (100.0)	-	
*Candida kefyr*	1 (2.7)	1 (100.0)	-	
*Candida krusei*	1(2.7)	1 (100.0)	-	
*Candida lusitaniae*	1 (2.7)	1 (100.0)	-	
*Candida* spp.	1 (2.7)	-	1 (100.0)	
**Neutropenia *n* (%)**				0.384 *
Yes	31 (88.6)	13 (44.8)	16 (55.2)	
No	6 (16.2)	-	6 (100)	
**On which day of neutropenia did Candida infection develop?** mean ± SD; median (minimum–maximum)	12.1 ± 14.8 7.0 (0–75)	8.6 ± 7.2 7.0 (0–30)	15.4 ± 19.3 9.0 (0–75)	0.628 *
**Disease period duration** mean ± SD; median (minimum–maksimum)	15.3 ± 11.3 12.0 (0–42)	17.3 ± 12.0 14.0 (0–42)	14.7 ± 11.7 11.0 (0–38)	0.431 *
**Concomitant bacteremia**				
No	31 (83.7)	10 (40.0)	15 (60.0)	1.000 **
Yes	6 (16.2)	3 (50.0)	3 (50.0)	
Gram (+)	3	2	1	0.400
Gram (−)	*3*	*-*	*3*	
**Local infections *n*(%)**				
No	32 (86.5)	11 (39.3)	17 (60.7)	1.000 **
Yes	5 (13.5)	2 (40.0)	3 (60.0)	
Lung infection	2	1	1	0.404 **
Hepatosplenic Candidiasis	1	1	-	
Scrotal lesion	1	-	1	
Skin infection on back	1	-	1	
**Antifungal use *n* (%)**				
Non	7 (18.9)	3 (50.0)	3 (50.0)	0.402 **
Single	19 (51.4)	5 (31.3)	11 (68.8)	
Double	7 (18.9)	3 (37.5)	5 (62.5)	
More than two	3 (8.1)	2 (66.7)	1 (33.3)	
Two antifungals in succession	1 (2.7)	-	1 (100.0)	
Amphotericin B	1	1	-	0.180
Fluconazolel	6	-	5	
Caspofungin	10	4	4	
Voriconazole	2	-	2	
Fluconazole + Amphotericin B	1	-	1	
Fluconazole + Caspofungin	4	1	3	
Fluconazole and Caspofungin sequentially	1	-	1	
Kaspofungin + Amphotericin B	1	-	1	
Kaspofungin + Micafungin	1	1	-	
Fluconazolel + Caspofungin + Micafungin	1	1	-	
Fluconazole + Caspofungin + Voriconazole	1	1	-	
Fluconazolel + Caspofungin + Amphotericin B	1	-	1	

Percentages for the whole group are column percentages; percentages for the survival columns are row percentages calculated from cases that remain at follow-up and whose survival status is known. * Mann-Whitney U test, ** Fisher’s exact chi-square test, *** HRG: high-risk group, **** NonHRG: non-high-risk group.

**Table 2:** The antifungal susceptibilities of all *Candida* isolates and the antifungal susceptibilities according to *Candida* species.

*Antifungal Agent by Candida* Species	** MIC_50_ (µg/mL)	*** MIC_90_ (µg/mL)	MIC Range (µg/mL)	Susceptible *n* (%)	Dose-Dependent Susceptible *n* (%)	Resistant *n* (%)
***All Candida Isolates (n = 37)***						
Anidulafungin	0.15	2	0.015–2	33 (94.3)	-	2 (5.7)
Micafungin	1	2	0.002–2	29 (100.0)	-	-
Caspofungin	0.25	0.5	0.015–1	31 (100.0)	-	-
Flucytosine	0.06	0.12	<0.06–1	-	-	-
Posaconazole	0.015	0.06	0.008–0.25	28 (93.3)	-	2 (6.7)
Voriconazole	0.015	0.5	0.008–2	26 (86.7)	-	4 (13.3)
Itraconazole	0.06	0.12	<0.06–0.25	27 (96.4)	-	1 (3.6)
Fluconazole	0.5	128	0.12–256	27 (84.4)	1(3.1)	4 (12.5)
Amphotericin B	0.5	1	0.125–2	34 (97.1)	-	1 (2.9)
***Candida parapsilosis* complex (*n* = 16)**						
Anidulafungin	1	2	0.25–2	16 (100.0)	-	-
Micafungin	1	2	0.5–2	16 (100.0)	-	-
Caspofungin	0.25	0.5	0.12–1	16 (100.0)	-	-
Flucytosine	≤0.06	0.25	<0.06–64	-	-	-
Posaconazole	0.015	0.03	0.008–0.08	15 (93.8)	-	1 (6.2)
Voriconazole	0.015	0.5	0.008–2	13 (81.3)	-	3 (18.7)
Itraconazole	0.06	0.12	0.015–0.12	16 (100.0)	-	
Fluconazole	0.5	32	0.25–128	13 (81.3)	-	3 (18.7)
Amphotericin B	0.5	1	0.25–2	15 (93.8)	-	1 (6.2)
***Candida albicans* (*n* = 11)**						
Anidulafungin	0.06	0.15	0.015–2	10 (90.9)	-	1 (9.1)
Micafungin	0.015	0.15	0.02–2	11 (100.0)	-	-
Caspofungin	0.03	0.03	0.015–0.5	11 (100.0)	-	-
Flucytosine	0.06	0.12	<0.06–0.25			
Posaconazole	0.015	0.03	0.015–0.06	11 (100.0)	-	-
Voriconazole	0.008	0.015	0.008–0.03	11 (100.0)	-	-
Itraconazole	0.06	0.06	0.015–0.06	11 (100.0)	-	-
Fluconazole	0.25	1	0.12–1	11 (100.0)	-	-
Amphotericin B	0.5	1	0.125–1	11 (100.0)	-	-
***Candida haemulonii* complex (*n* = 3)**						
Anidulafungin	0.06	0.06	0.06	3 (100.0)	-	-
Micafungin	0.03	0.03	0.03			
Caspofungin	0.03	0.03	0.03			
Flucytosine	0.12	0.12	0.12			
Posaconazole	0.12	0.12	0.12			
Voriconazole	8	8	8.00			
Itraconazole	0.025	0.25	0.25			
Fluconazole	0.12	0.12	0.12			
Amphotericin B	0.25	0.25	0.25	3 (100.0)	-	-
***Candida tropicalis* (*n* = 2)**						
Anidulafungin	0. 06	0.5	0.06–0.5	1 (50.0)		1 (50.0)
Micafungin	0.03	0.5	0.03–0.5	-	-	-
Caspofungin	0.03	0.25	0.03–0.25	1 (50.0)	*	*
Flucytosine	0.06	0.12	0.06–0.12	-	-	-
Posaconazole	0.03	0.25	0.03–0.25	1 (50.0)	-	1 (50.0)
Voriconazole				1 (50.0)	-	1 (50.0)
Itraconazole	0.06	0.25	0.06–0.25	1 (50.0)	-	1 (50.0)
Fluconazole	0.5	2	0.5–2	2 (100.0)	-	-
Amphotericin B	0.5	1	0.50–1	2 (100.0)	-	-

* EUCAST recommends that isolates susceptible to Anidulafungin or Micafungin should also be considered susceptible to Caspofungin. One isolate was resistant to Anidulafungin and its susceptibility to Micafungin has not been determined. MIC: minimum inhibitory concentration. ** MIC_50_ and *** MIC_90_: MIC required to inhibit 50% and 90% of the isolates, respectively.

## Data Availability

All data used in this study can be obtained from the author upon request.
